# Genome-Wide Analysis of the *NAC* Gene Family in Physic Nut (*Jatropha curcas* L.)

**DOI:** 10.1371/journal.pone.0131890

**Published:** 2015-06-30

**Authors:** Zhenying Wu, Xueqin Xu, Wangdan Xiong, Pingzhi Wu, Yaping Chen, Meiru Li, Guojiang Wu, Huawu Jiang

**Affiliations:** 1 Key Laboratory of Plant Resources Conservation and Sustainable Utilization, South China Botanical Garden, Chinese Academy of Sciences, Guangzhou 510650, China; 2 University of Chinese Academy of Sciences, Beijing 100049, China; Institute of Genetics and Developmental Biology, Chinese Academy of Sciences, CHINA

## Abstract

The NAC proteins (NAM, ATAF1/2 and CUC2) are plant-specific transcriptional regulators that have a conserved NAM domain in the N-terminus. They are involved in various biological processes, including both biotic and abiotic stress responses. In the present study, a total of 100 *NAC* genes (*JcNAC*) were identified in physic nut (*Jatropha curcas* L.). Based on phylogenetic analysis and gene structures, 83 *JcNAC* genes were classified as members of, or proposed to be diverged from, 39 previously predicted orthologous groups (OGs) of NAC sequences. Physic nut has a single intron-containing *NAC* gene subfamily that has been lost in many plants. The *JcNAC* genes are non-randomly distributed across the 11 linkage groups of the physic nut genome, and appear to be preferentially retained duplicates that arose from both ancient and recent duplication events. Digital gene expression analysis indicates that some of the *JcNAC* genes have tissue-specific expression profiles (e.g. in leaves, roots, stem cortex or seeds), and 29 genes differentially respond to abiotic stresses (drought, salinity, phosphorus deficiency and nitrogen deficiency). Our results will be helpful for further functional analysis of the *NAC* genes in physic nut.

## Introduction

The NAC proteins are one of the largest groups of plant-specific transcriptional regulators, which share a conserved N-terminus DNA binding domain originally found in four genes: *ATAF1/ATAF2* and *Cup-Shaped Cotyledon2* (*CUC2*) in *Arabidopsis* and *No Apical Meristem* (*NAM*) in *Petunia hybrida*. In petunia, NAM is required for pattern formation in embryos and flowers and expressed at meristem and primordia boundaries [1]. *Arabidopsis* CUC1 and CUC2 are involved in the formation of embryo, floral organs and shoot apical meristems [2]. ATAF1 and ATAF2 are reported to respond to biotic and abiotic stress in *Arabidopsis* [3, 4]. NAC proteins contain a NAM domain consisting of five conserved subdomains at the N-terminus (designated A-E) [5, 6]. Subdomains A, C and D are highly conserved, while B and E are more variable [7].

NAC proteins have been found to participate in diverse plant developmental processes, including apical meristem development [1, 8], organ formation and development [9], leaf senescence [10], fruit ripening [11], hormone signaling [12], and both biotic and abiotic stress responses [12, 13]. Accordingly, large numbers of putative *NAC* genes have been identified, including 117 in *Arabidopsis* and 151 in rice (*Oryza sativa* L.) [14], 74 in grape (*Vitis vinifera*) [15], 152 in soybean (*Glycine max* L.) [16], 88 in pigeonpea (*Cajanus cajan* (L.) Millsp.) [17], 147 in foxtail millet (*Setaria italica* L.) [18], and 204 in Chinese cabbage (*Brassica pekinensis*) [19]. Comprehensive analysis of the NAC family genes in rice and *Arabidopsis* has divided the NAC proteins into two large groups (Groups I and II), and sequences of the NAC domain in Group I, but not in Group II are highly conserved [7]. In addition, based on an expert NAC sequence comparison, 40 orthologous groups (OGs) of NAC sequences were proposed to be probably derived from an ancestral gene which presents in the most recent common ancestor of dicots and monocots [20].

Physic nut (*Jatropha curcas* L.) is a perennial plant of the Euphorbiaceae family that has received great attention as a renewable resource for biodiesel production. It has attractive features for its high resistance to various stresses, including drought and nutrient deficiencies [21]. Thus, its transcriptional responses to several stresses, including cold [22] and salinity [23], have been examined in attempts to elucidate the mechanisms involved in and (potentially) extend its resistance. However, the roles of NAC proteins in physic nut have not yet been explored, although they play key roles in the formation of plants’ organs [9] and stress responses [12, 13].

Genomic sequence and expressed sequence tags (ESTs) libraries that we and others have recently constructed [24–26] provide convenience for us to analyze the gene families and their evolution in physic nut. For example, 58 *WRKY* genes were identified in the physic nut genome, and no evidence of recent gene duplication was detected in a phylogenetic comparison of *JcWRKY* genes and those of *Arabidopsis*, rice and castor bean [27]. In the study presented here, we identified the *NAC* genes of physic nut (*JcNACs*) from genome sequences. We then characterized the overall exon/intron arrangements of the full genes and the conserved NAC domain-coding sequences. The distribution of these genes in the linkage groups (LGs) and a phylogenetic tree combining castor bean, *Arabidopsis* and rice NAC proteins were also analyzed to examine their evolutionary relationships and the putative functions of physic nut NAC proteins. Finally, we analyzed the expression patterns of *JcNAC* genes under both normal growth conditions and abiotic stresses.

## Materials and Methods

### Ethics statement

No specific permits were required for the field studies described because the experiment site is owned by South China Botanical Garden, Chinese Academy of Sciences, and the Key Laboratory of Plant Resources Conservation and Sustainable Utilization undertook the management. No specific permits were required for these locations/activities, because the location is not privately-owned or protected in any way and the field studies did not involve endangered or protected species.

### Sequence retrieval and identification

Sequences of *Arabidopsis* NAC proteins were retrieved from the *Arabidopsis* genome database, TAIR 9.0 (http://www.arabidopsis.org/), and used as queries in BLAST searches against the physic nut genome database of the KaZuSa DNA Research Institute (http://www.kazusa.or.jp/jatropha/) [24] and our own genome database, which is available from DDBJ/EMBL/GenBank under Accession no. AFEW00000000 (the version described in this paper is the first version, AFEW01000000). Sequences were selected for further analysis if the E value was less than 1e^-10^. Next, we corrected errors in annotation of NAC coding sequences on the basis of the physic nut EST database available from GenBank (http://www.ncbi.nlm.nih.gov/) and our own physic nut and *Jatropha integerrima* (allied species of physic nut) EST datasets (SRA197144 and SRA197148 in GenBank). Finally, all putative proteins were confirmed to be NAC proteins by the Pfam program (http://pfam.xfam.org/) and the confirmed sequences were aligned using Clustal X to remove redundant sequences. In addition, length, molecular weight and isoelectric point parameters of each JcNAC protein were calculated by the online ExPasy program (http://www.expasy.org/tools/) [28].

### Chromosomal localization of the *JcNAC* genes

Chromosome localization was completed using MapChart 2.1 [29] based on the linkage map we have constructed [26]. Firstly, the specific positions of each *JcNAC* gene on corresponding scaffold were obtained from our genome database. Secondly, the detailed locations of each *JcNAC* gene were calculated based on the adjacent mapping markers located on our genetic-linkage map. Lastly, chromosome localization was completed using MapChart 2.1. Tandem repeats were defined as repeats located within 50 kb of each other, or separated by less than 4 non-homologous spacer genes [30].

### Phylogenetic analysis of the *NAC* gene family

To analyze relationships of the *NAC* genes in physic nut, we used putative JcNAC proteins and NAC proteins from another three species to construct a phylogenetic tree: Arabidopsis (dicotyledon), rice (monocotyledon) and castor bean (dicotyledon, Euphorbiaceae). Sequences of the rice NAC proteins were downloaded from the rice genome annotation database (http://rice.plantbiology.msu.edu/, release 5.0). Castor bean NAC proteins were obtained from Phytozome (http://www.phytozome.net) [31]. Proteins of 28229.m000059, 28492.m000489, 30076.m004482 and 30157.m000796 were not used for alignments and phylogenetic analysis because sequences of their NAC domains are incomplete. Multiple sequence alignments of the conserved NAC domain sequences were performed using Clustal W. Unrooted maximum-likelihood (ML) trees were constructed using the LG model with aLRT SH-like branch support steps in PhyML version 3.0 (http://atgc.lirmm.fr/phyml/) [32].

### Gene structure analysis and conserved motif composition prediction

The exon/intron structures of *JcNAC* genes were determined by comparing the coding sequences and corresponding genomic sequences in the Gene Structure Display Server (GSDS, http://gsds.cbi.pku.edu.cn/) [33]. Conserved motifs were analyzed using the MEME program (http://meme.nbcr.net/meme/) [34]. Transmembrane helices in JcNAC proteins were predicted using TMHMM Server v. 2.0 (http://www.cbs.dtu.dk/services/TMHMM-2.0/) [35]. The *cis*-elements analysis was performed in the promoter sequences (1 kb upstream region) of the *JcNAC* coding main sequences using the program MEME [34] and PLACE (A Database of Plant *cis*-acting Regulatory DNA Elements, http://www.dna.affrc.go.jp/PLACE/signalscan.html) [36]. The MEME program was used to search new putative *cis*-motif consensus patterns of 8–12 bases width, with E-value less than 0.01, only on the forward strand of the input sequences. The PLACE program was used to search the known *cis*-acting elements reported in the previous studies, which commonly existed in all of the corresponding *JcNAC* genes’ promoters.

### Preparation of plant materials

After disinfection with 1:5000 KMnO_4_ solution, seeds of the inbred physic nut (*J*. *curcas* L.) cultivar GZQX0401 were planted in sand to germinate. When cotyledons were fully expanded, seedlings were transferred to trays containing a 3:1 mixture of sand and soil soaked with half-strength Hoagland solution in a greenhouse (30–35°C) illuminated with natural sunlight (100–300 μmol m^-2^ s^-1^) in Guangzhou (113.3° E, 23.1° N). After emergence of the first true leaf, the trays were irrigated with 1 liter of Hoagland nutrient solution (pH 6.0) once every two days at dusk. The Pi and N deficiency treatment was begun at the six-leaf stage (eight weeks after germination), after removing most nutrients by five washes with 1 liter of tap water. The plants assigned to the P- or N- deficiency treatments were then irrigated daily with Hoagland nutrient solution minus phosphorus or minus nitrogen). For the salinity treatment, the seedlings were irrigated with Hoagland solution plus 100 mM NaCl every day. For the drought treatment, irrigation was withheld. Roots were sampled after 1 day, 4 days and 7 days of drought stress; after 2 hours, 2 days and 7 days of salinity stress; and after 2 hours and 2 days of phosphorus and nitrogen deficiency. Samples were frozen immediately in liquid nitrogen and stored at -80°C prior to quantitative PCR (qRT-PCR) analysis.

### RNA isolation and qRT-PCR analysis

Total RNA of roots was extracted using the CTAB method with some modifications [37]. Briefly, the CTAB extraction buffer was spermidine-free, and 1/4 volume 5 M KAc (pH 4.8) was added before the first centrifugation; 1/4 volume 8 M LiCl was used for precipitation; and the pellet was dissolved in guanidine thiocyanate solution (2.5 g guanidine thiocyanate in 6.6 mL CBS solution (42 mM sodium citrate and 8.38 g L^-1^ N-lauroylsarcosine sodium) instead of SSTE. Subsequently, first-strand cDNA was synthesized from 2 μg of total RNA treated with RNase-Free DNase I (Roche), using M-MLV reverse transcriptase (Promega) according to the manufacturer’s instructions, and qRT-PCR analysis was performed using a physic nut *Actin* gene as an internal control. The specific primer sequences and the PCR reaction conditions are listed in S1 Table. A LCS480 system (Roche) was used for all qRT-PCR assays. Each 20 μL reaction volume included 10 μL of twofold SYBR Premix ExTaq, 0.4 μL of forward and reverse primer (10 μmol), 2 μL of diluted cDNA solution, and 7.2 μL of ddH_2_O The thermal profile used for all PCR amplifications was: 10 min at 95°C for DNA polymerase activation, followed by 40 cycles of 15 s at 95°C and 35 s at 60°C. The expression levels were calculated using the 2^-ΔΔCT^ method. Each PCR assay was run in triplicate for two independent biological repeats.

## Results

### Identification and characterization of *JcNAC* genes

Based on the domain sequences of the NAC family members of *Arabidopsis* and rice, a total of 100 NAC family members were identified both from the publicly available genome database [24] and our physic nut database (DDBJ/EMBL/GenBank under accession no. AFEW00000000) [26] using the BlastP and tBlastn programs. Next, we corrected errors in several annotated NAC coding sequences according to the available EST sequences for physic nut in GenBank and our physic nut and *J*. *integerrima* L. EST database (SRA197144 and SRA197148 in GenBank). We designated JcNAC001 to JcNAC100 according to the phylogenetic tree constructed from alignments of predicted NAC proteins, and their accession numbers in GenBank, and parameters of the predicted proteins, are listed in S2 Table. According to the detailed information, lengths of these predicted JcNAC proteins ranged from 147 (JcNAC070) to 709 residues (JcNAC066), with an average of 340 aa, and the isoelectric point (PI) ranged from 4.56 (JcNAC077) to 9.56 (JcNAC060). To analyze the NAM domain of these predicted JcNAC proteins, the Pfam program was used and the results indicated that they indeed contained a conserved NAM domain (pfam02365/cl03558) [5, 6] at the N-terminus of the amino acid sequences (S2 Table).

**Fig 1:**
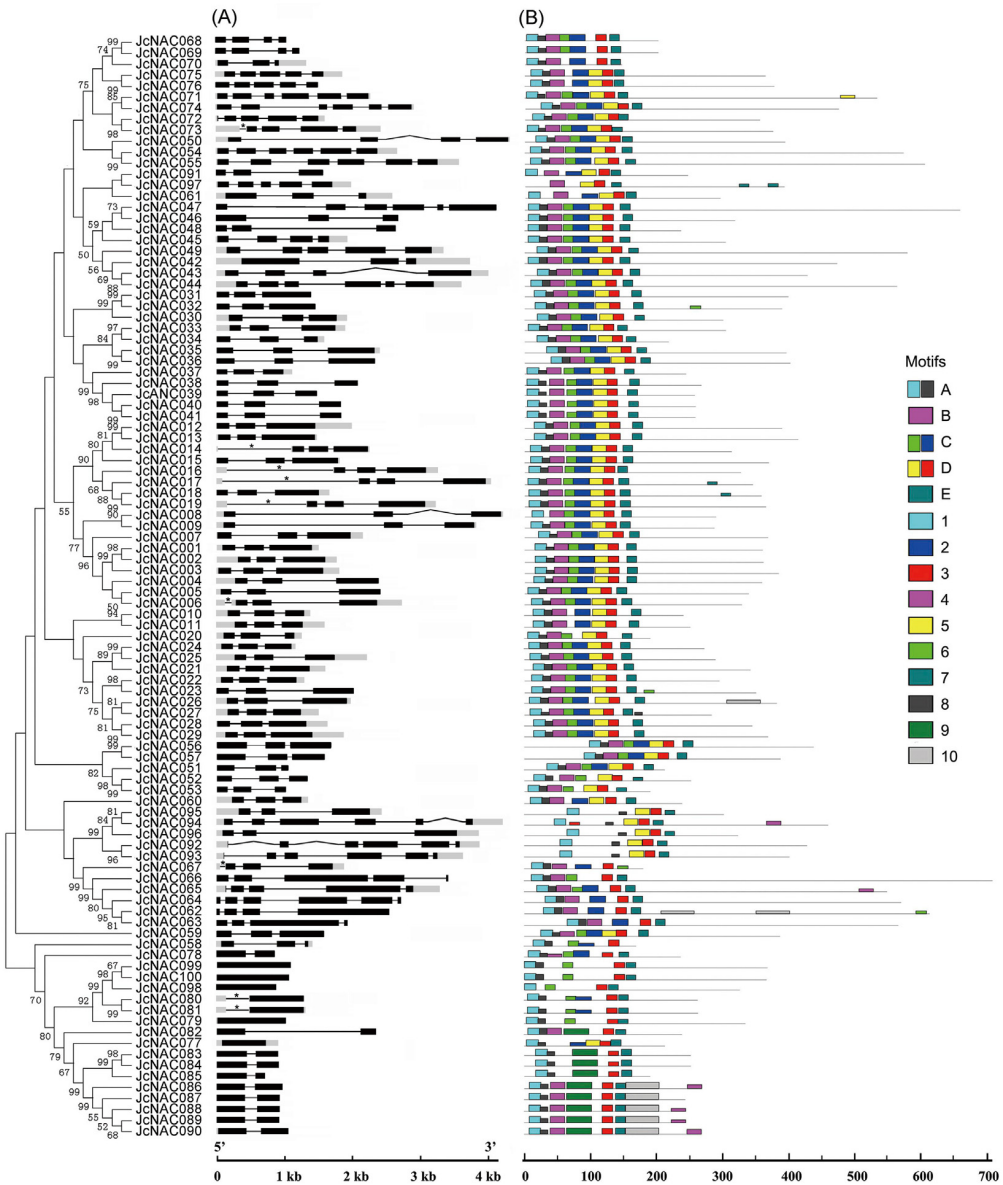
Gene structure and motif locations of *JcNAC* genes. (A), Exon/intron arrangements of *JcNAC* genes. Exons and introns are represented by black boxes (open reading frame in black, untranslated region (UTR) in gray), and black lines, respectively, and their sizes are indicated by the scale at the bottom. *, intron in the UTR. (B), Schematic representation of conserved motifs in the JcNAC proteins predicted by MEME. Each motif is represented by a number in the colored box. The black lines represent non-conserved sequences. The relationships between motifs and conserved domains (A-E) are shown on the right. The lengths of JcNAC proteins can be estimated from the scale at the bottom.

Several NAC proteins have been previously shown to own a membrane-bound domain [38]. In this study, the membrane-bound domain of all the JcNAC proteins was analyzed using the TMHMM server v. 2.0. Eight JcNAC proteins (JcNAC044, 047, 049, 050, 054, 055, 071 and 074) were observed to contain transmembrane amino acid sequences in their C-terminus (S2 Table). Analysis of exon/intron structures displayed that 58 *JcNAC* genes have two introns, 10 have one intron, 10 have five introns, 8 have three introns, 7 have four introns, and 7 genes are intronless within coding sequences in their ORF (open reading frame). Nine genes have intron in the untranslated region (Fig 1A).

### Conserved motifs of the JcNAC proteins

Analysis of the sequence features of JcNAC proteins using MEME [34] predicted 10 conserved motifs (S1 Fig). Amino acid sequences of motifs 1 plus 8, 4, 6 plus 2, 5 plus 3, and 7 respectively correspond to the five well-conserved subdomains (A-E) of the NAC domain [39]. Motif 9 is similar to motif 6 plus 2, composing subdomain C (Fig 1B). The amino acid sequences of these motifs are highly conserved, like homologous sequences detected in NAC proteins from *Arabidopsis*, pigeonpea, rice, and *Populus* [7, 17, 40]. Motif 10 corresponds to a C-terminus transcription regulatory region (TRR). About 60% of the predicted JcNAC proteins contain all five characteristic motifs of the NAC domain, while the others appear to have lost one or more of these motifs.

The exon/intron structures of NAC domain coding sequences have been shown to be conserved in grape, *Arabidopsis*, rice, and banana (*Musa acuminate*) *NAC* genes, and the intron-containing genes have been classified into nine models in the previous studies [15, 20]. Analysis of the overall exon/intron arrangements showed that intron-containing *NAC* genes from physic nut could be grouped into these model structures of NAC domain-coding sequences as well (S2 Table). The NAC domain of 73 intron-containing genes in physic nut was encoded by the first three exons: the first encoding the subdomains A and B, the second encoding the subdomains C and D, and the third encoding the subdomain E (S2 Table). Among these members, *JcNAC046* and *JcNAC061* have lost the first intron, while *JcNAC042* has lost both introns. The NAC domain in JcNAC062-065 is encoded by the second to the fourth exons (the intron between the third and fourth exons is missing in *JcNAC063* and *067*). While in JcNAC094-096, it is encoded by the second and third exons, and the second exon encodes the subdomains A and B, while the third exon encodes the subdomains C-E. In JcNAC092 and 093, it is encoded by the third and fourth exons. In the single intron-containing genes, *JcNAC078* and *JcNAC082*-*090*, the first exon encodes the subdomains A-D, and the second exon encodes the subdomain E (S2 Table).

### Phylogenetic analysis of NAC proteins

The previous phylogenetic analysis has classified the NAC family genes into two major groups, Groups I and II, and those of Group I (but not Group II) are highly conserved [7]. Group I and some Group II members have been further classified into 40 orthologous groups (OGs) for dicots and monocots [20]. To investigate the phylogenetic relationships among the *NAC* genes in physic nut and other plants, a Maximum Likelihood phylogenetic tree was constructed in PhyML version 3.0 [32] from alignments of the conserved NAC domain sequences of all predicted NAC proteins from physic nut (dicot, Euphorbiaceae) and castor bean (dicot, Euphorbiaceae), and partial NAC proteins (including all proteins that had been classified into OGs) [20] from rice (monocot) and *Arabidopsis* (dicot) (S2 Fig). This phylogenetic tree was perfectly consistent with the determined OGs of *NAC* genes for grape, *Arabidopsis*, rice and banana [20] when compared the genes from *Arabidopsis* and rice in each of the subclusters. The phylogenetic tree clarified that these NAC proteins could be divided into the 6 main clusters (CL00002–00007) determined by Cenci et al. [20]. So, we also performed six additional phylogenetic analyses with the orthologous genes of each main cluster to verify their phylogenetic relationship (**Panels A-F in** S3 Fig). Based on these phylogenetic trees and their gene structures, 60 *JcNAC* genes were classified into the proposed 39 OGs of dicot NACs [20] with aLRT values spanning between 0.600 and 0.999 (except OG2f) (Table 1; S3 Table and **Panels A-F in** S3 Fig). Physic nut has multiple copies of genes in 14 of the 39 OGs. Among the 37 OGs which contain both dicot and rice genes, 30 showed the expected dicot and rice clustering in these phylogenetic analyses. ANAC105 in OG2b, ANAC045 and ANAC086 in OG4a, ANAC013 in OG4g, and 29992.m001406 in OG7e occupy unexpected positions. Three OGs (2a, 3c, and 5a) should contain gene duplications that occurred before the dicot and rice divergence.

**Table 1:** Distribution of physic nut *NAC* genes in orthologous groups (OGs).

OGs	Genes	OGs	Genes	OGs	Genes	OGs	Genes
**1a**	*JcNAC009*	**3d**	*JcNAC029*	**5bL**	*JcNAC064*	**8a**	*JcNAC034*
**1b**	*JcNAC008*	**3e**	*JcNAC021*		*JcNAC065*	**8b**	*JcNAC037*
**1c**	*JcNAC006*	**3L**	*JcNAC051*		*JcNAC066*		*JcNAC038*
**1d**	*JcNAC004*		*JcNAC052*		*JcNAC067*		*JcNAC039*
**1e**	*JcNAC007*		*JcNAC053*		*JcNAC068*		*JcNAC040*
**1f**	*JcNAC005*		*JcNAC056*		*JcNAC069*		*JcNAC041*
**1g**	*JcNAC003*		*JcNAC057*		*JcNAC070*	**SI**	*JcNAC078*
**1h**	*JcNAC001*		*JcNAC059*		*JcNAC071*		*JcNAC082*
	*JcNAC002*	**4a**	*JcNAC047*		*JcNAC072*		*JcNAC083*
**1L**	*JcNAC091*	**4b**	*JcNAC048*		*JcNAC073*		*JcNAC084*
**2a**	*JcNAC018*	**4c**	*JcNAC045*		*JcNAC074*		*JcNAC085*
	*JcNAC019*	**4d**	*JcNAC043*		*JcNAC075*		*JcNAC086*
**2b**	*JcNAC017*		*JcNAC044*		*JcNAC076*		*JcNAC087*
**2c**	*JcNAC016*	**4e**	*JcNAC042*	**6a**	*JcNAC035*		*JcNAC088*
**2d**	*JcNAC015*	**4f**	*JcNAC046*		*JcNAC036*		*JcNAC089*
**2e**	*JcNAC014*	**4g**	*JcNAC049*	**6b**	*JcNAC031*		*JcNAC090*
**2f**	*JcNAC012*	**5a**	*JcNAC010*		*JcNAC032*	**IL1**	*JcNAC077*
	*JcNAC013*		*JcNAC011*	**6c**	*JcNAC030*	**IL2**	*JcNAC079*
**3a**	*JcNAC024*		*JcNAC060*	**7a**	*JcNAC095*		*JcNAC080*
	*JcNAC025*		*JcNAC058*		*JcNAC096*		*JcNAC081*
**3b**	no	**5b**	*JcNAC054*	**7b**	*JcNAC094*		*JcNAC098*
**3c**	*JcNAC022*		*JcNAC055*	**7c**	*JcNAC092*		*JcNAC099*
	*JcNAC023*	**5c**	*JcNAC050*	**7d**	*JcNAC093*		*JcNAC100*
	*JcNAC026*	**4/5L**	*JcNAC061*	**7e**	*JcNAC020*		
	*JcNAC027*	**5bL**	*JcNAC062*	**7f**	*JcNAC097*		
**3d**	*JcNAC028*		*JcNAC063*	**8a**	*JcNAC033*		

Six *JcNAC* genes close to sequences of cluster 3 were classified as OG3-like (3L). These proteins share higher amino acid identities to OG3 proteins than other OG proteins, and they are encoded by two-intron containing genes. Fifteen *JcNAC* genes close to cluster 5b were classified as 5bL. These genes have variable numbers of exons and introns, but their NAC domains share the highest amino acid identities with OG5 proteins, and are located in three exons (except for *JcNAC063* and *067*, which have lost the second intron). Two (JcNAC071 and 074) have a membrane-bound domain in the C-terminal region, which is the feature of several OGs4 and OGs5 proteins. Single intron containing genes (*JcNAC078* and *082*–*090*) were classified as SI, which have no detected orthologous genes in the castor bean genome.

The intronless (IL) genes were classed into two groups, named IL1 and IL2 (Table 1 and S3 Table). Proteins in the SI, IL1, and IL2 groups appeared to be very divergent from the other NACs. JcNAC061 and JcNAC091 also appeared to be very divergent from the other NACs. Based on the exon/intron structures, *JcNAC061* and its orthologs (*XP_002282899* in grape and *30170*.*m013849* in castor bean) have two introns in their CDS, and NAC subdomains A-D are encoded by the first exon, suggesting that these genes have lost the first intron which present in most *NAC* genes. Their gene structures are similar to partial genes of OGs 4 and 5, so we named them as 4/5L. *JcNAC091* was classified as 1L, based on the phylogenetic analysis and exon/intron structures, which is similar to OG1 genes (Table 1 and S2 Fig).

To examine the phylogenetic relationship among the *NAC* genes those were not classified into the 40 OGs in physic nut, *Arabidopsis* and rice, an unrooted tree was constructed from alignments of the NAC domain sequences of these proteins (**Panel G in** S3 Fig). Based on this phylogenetic tree, the 5bL subgroup of physic nut was clustered into a group with the ANAC001 subgroup of *Arabidopsis* [7]. The intronless *NAC* genes from rice and *Arabidopsis*, and the IL and SI genes from physic nut were clustered into species specific subgroups, which indicated that they are expanded within different linkages and highly divergent from each other. The other genes contain one or two introns within their NAC domain coding sequences. The amino acid sequences of their NAC domains between different clades are highly divergent with low identities less than 40%.

### Chromosomal distribution and duplication analysis of *JcNAC* genes

**Fig 2:**
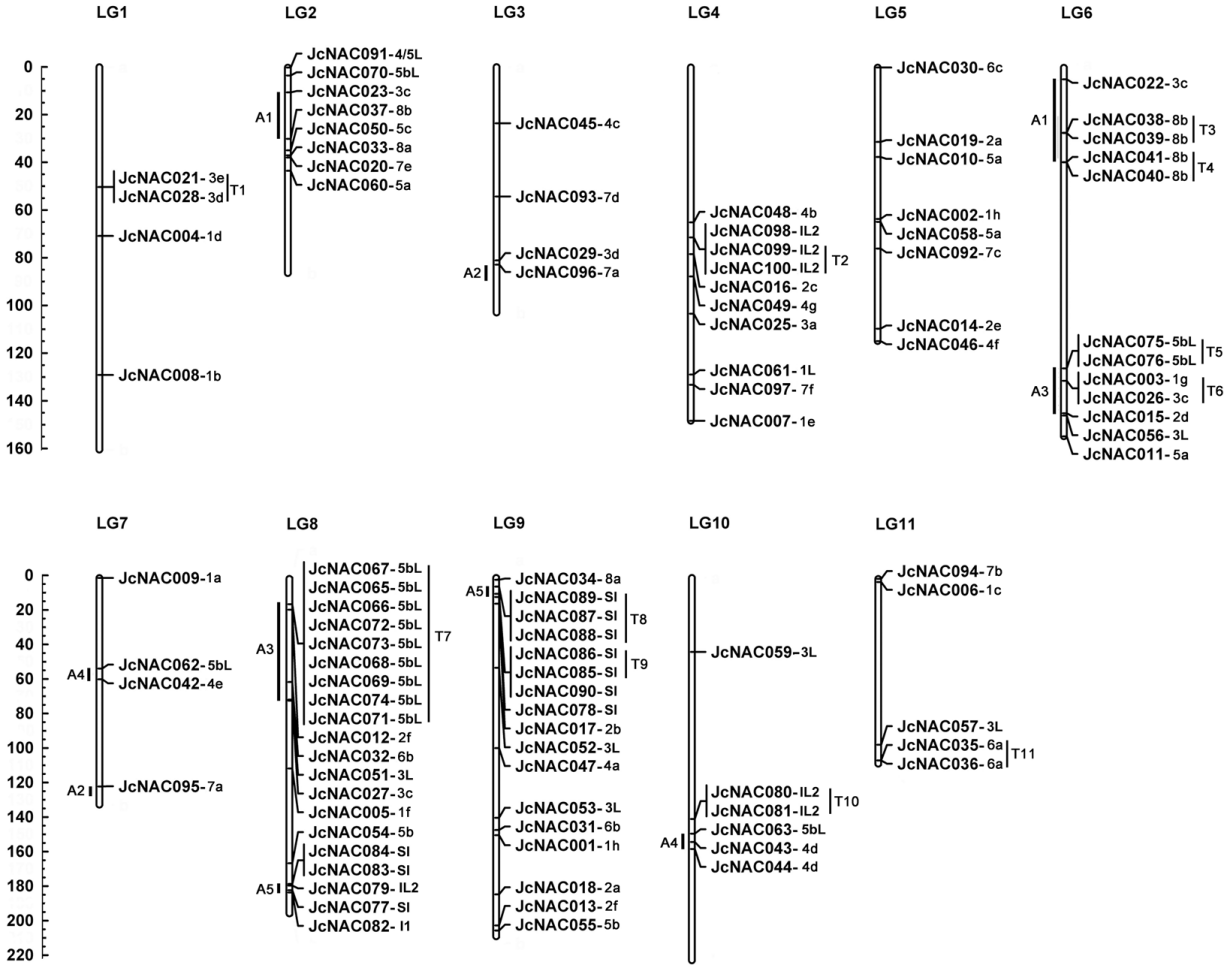
Chromosomal localization of physic nut *NAC* genes. Chromosomal localization of *JcNAC* genes based on the linkage map. In total, 98 *JcNAC* genes were mapped to the 11 linkage groups (LGs). The scale is in centimorgan. T, tandem duplication; A, ancient segmental duplication based on the genome synteny [26].

**Fig 3:**
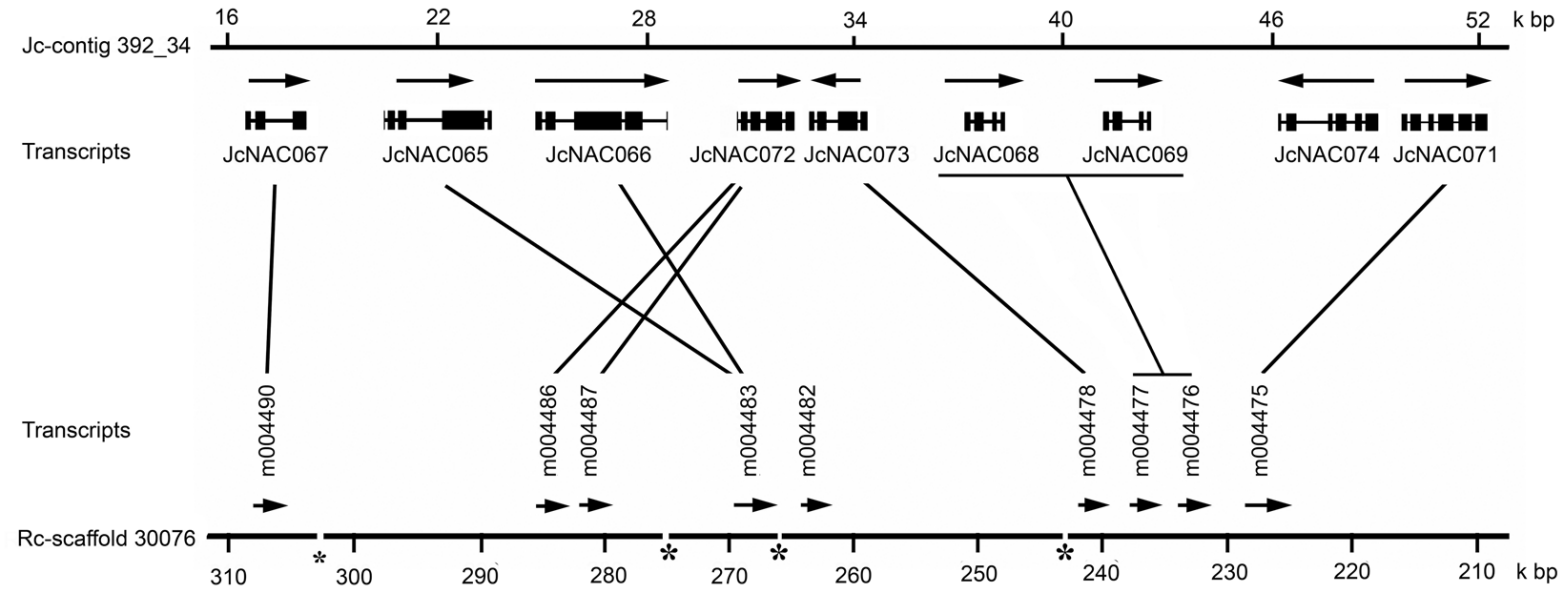
Synteny between physic nut and castor bean genomic segments containing *NAC* gene clusters. Nine genes in tandem repeats 7 (T7) on Jc-contig 392_34 of physic nut and their orthologs on scaffold 30076 of castor bean were syntenic. The NAC domain of the 30076.m004482 protein is incomplete. The orthologous genes based on the phylogenetic analysis (S2 Fig) were indicated by lines.

A total of 98 *JcNAC* genes were mapped to 11 chromosomes according to the linkage map we constructed [26], while the other two genes (*JcNAC024* and *064*) located on unmapped scaffolds (S2 Table). The distribution and density of the *JcNAC* genes on chromosomes were not uniform (Fig 2). Chromosome 8 contained the highest frequency (21%) of *JcNAC* genes, while chromosome 1, 3 and 5 contained the lowest frequencies (4%). Evidence of both segmental and tandem duplication events of *NAC* genes in plants has been previously reported [14–16, 40]. To determine the possible relationships between the *NAC* genes and potential segmental duplications, we mapped the *JcNAC* genes to the duplicated blocks established in the recent study [26]. As a result, five blocks of pairs (A1-A5) of *JcNAC* genes were observed that may have arisen from the corresponding duplicate genomic blocks (Fig 2). In addition, a total of 30 *JcNAC* genes (30% of the total number) were observed to be in tandem repeats genes, which we designated them as T1-T11 (Fig 2). Nine direct tandem repeats (*JcNAC065*-*069* and *071*–*074*) (T7) were observed on chromosome 8, which has the highest number of tandem repeats of *JcNAC* genes. Their orthologs in castor bean were also clustered on a genomic sequence of scaffold 30076 (Fig 3). According to the phylogenetic tree, T4, T8, T9, and T11 genes duplicated after separation of lineages of the analyzed species; T6 genes (*JcNAC003* and *026*, originating from OGs1g and 3c) on chromosome 6 arose from ancient tandem repeats, predating the divergence between monocots and dicots [20]; and T1 genes on chromosome 6 (*JcNAC021* and *028*, originating from OGs 3e and 3d) predate the divergence of dicots because homologous repeats are present in genomes of grape (*VvNAC17*/*GSVIVT01014403001* and *VvNAC17*/*GSVIVT01014405001*) and *Arabidospis* (*ANAC018*/*AT1G52880* and *ANAC019*/*AT1G52890*).

### Analysis of *JcNAC* genes’ expression patterns

**Fig 4:**
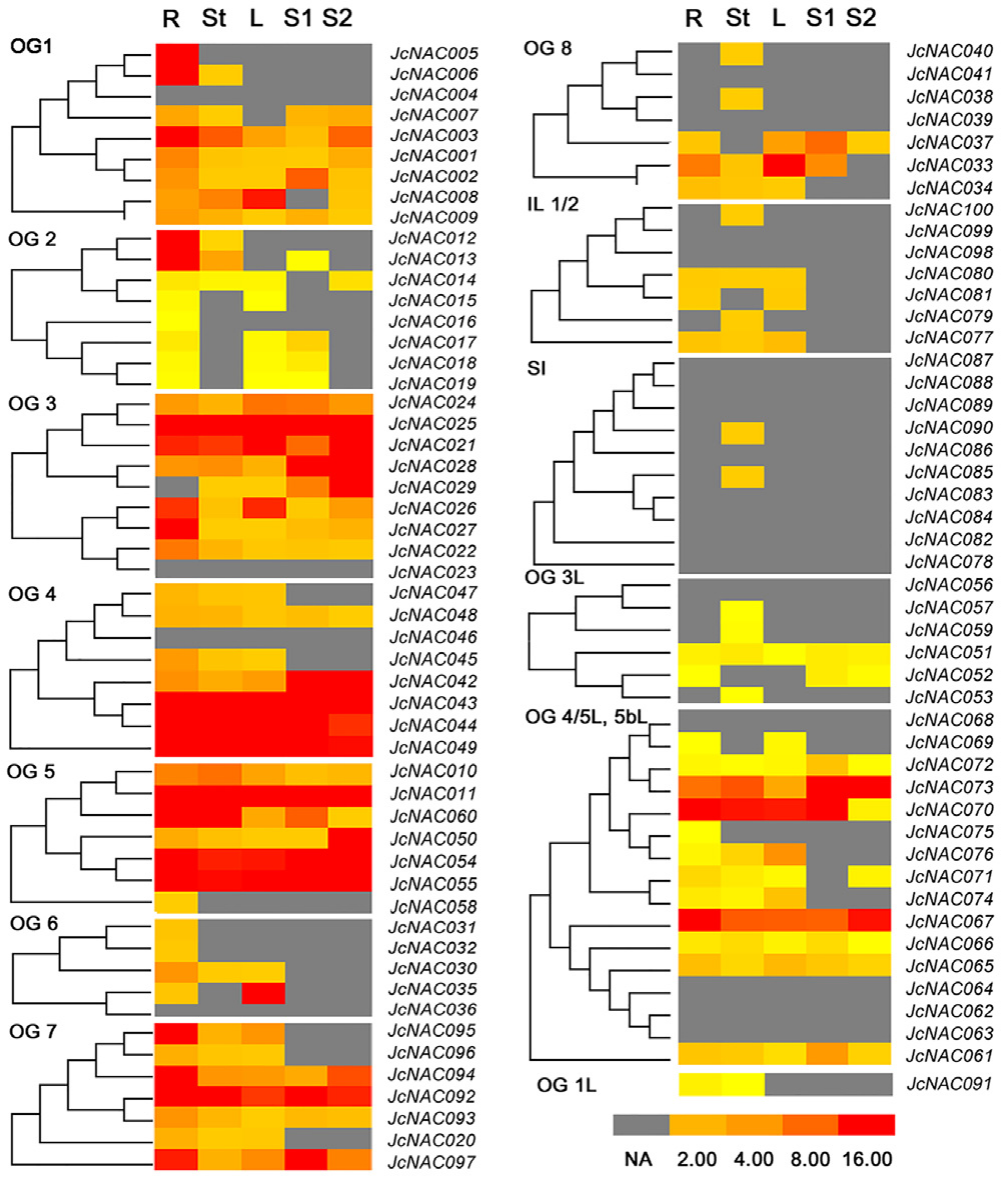
Relative expression levels of *JcNAC* genes, divided into different orthologous groups. The relative expression levels of all of the *JcNAC* genes were the average value of 6 biological repeats of digital expression profile tags. NA, not available; R, roots; St, stem cortex; L, leaves; S1, seed 1; S2, seed 2.

After analyzing the EST (Expressed Sequence Tags) databases of physic nut and/or its related species *J*. *integerrima*, ESTs representing 80 of the 100 *JcNAC* genes were detected. Weak expression of six other *JcNAC* genes was detected in our transcriptome expression database, generated using next-generation sequencing-based digital gene expression tags (S4 Table). Thus, 86 of the 100 predicted genes (86% of the total) were expressed in at least one of the tested tissues. To analyze the expression patterns of *JcNAC* genes, we retrieved information on expression levels of *JcNAC* genes under normal growth conditions in four tissues: roots, stems (shoot cortex), leaves, and seeds (in the early development stage, S1, and filling and maturation stage, S2) [41]. Eleven of these genes were expressed in all tested tissues at relatively high levels (greater than or equal to 10 transcripts per million tags, TPM), namely *JcNAC011*, *021*, *025*, *043*, *044*, *049*, *054*, *055*, *067*, *073*, and *092*. However, many *JcNAC* genes were found to be expressed at different levels in different tissues according to the digital gene expression tags (Fig 4). In developing seeds, expression levels of five genes (*JcNAC003*, *021*, *029*, *050* and *094*) were three-fold higher in the filling and maturation stage (S2) than in the early development stage (S1), while at least nine genes (*JcNAC002*, *011*, *033*, *037*, *054*, *060*, *070*, *092* and *097*) were more strongly expressed at S1 than at S2 (S4 Table). Most of the single intron (SI) genes did not show any credible expression based on EST and digital gene expression tags in the above tissues in physic nut (Fig 4). ESTs of *JcNAC082*, *083*, *087* and *090* orthologs were detected at high frequency in the *J*. *integerrima* flower cDNA library.

Several duplicators from genome triplication events in ancient dicotyledons were differently expressed among the tested tissues. For genes on the A1 locus, the middle expression level of *JcNAC022* (OG3c) in roots and *JcNAC037* (OG8b) in S1 were observed, whereas *JcNAC023* (OG3c) and *JcNAC038-040* (OG8b) genes were not detected in the tested tissues. The *JcNAC095* gene (OG7a) but not the *JcNAC096* gene (OG7a) on locus A2 was highly expressed in roots. The *JcNAC026* gene (OG3c) on locus A3 was highly expressed in roots and leaves, while the *JcNAC027* gene (OG3c) showed high expression level only in roots. For the tandem duplicators, the EST and high expression level of *JcNAC035* (OG6a) were observed in roots, whereas *JcNAC036* (OG6a) was not detected in the tissues tested on locus T11. For the 9 OG5bL genes on locus T7, the high expression level in the tested tissues was only observed for the *JcNAC073* gene.

**Fig 5:**
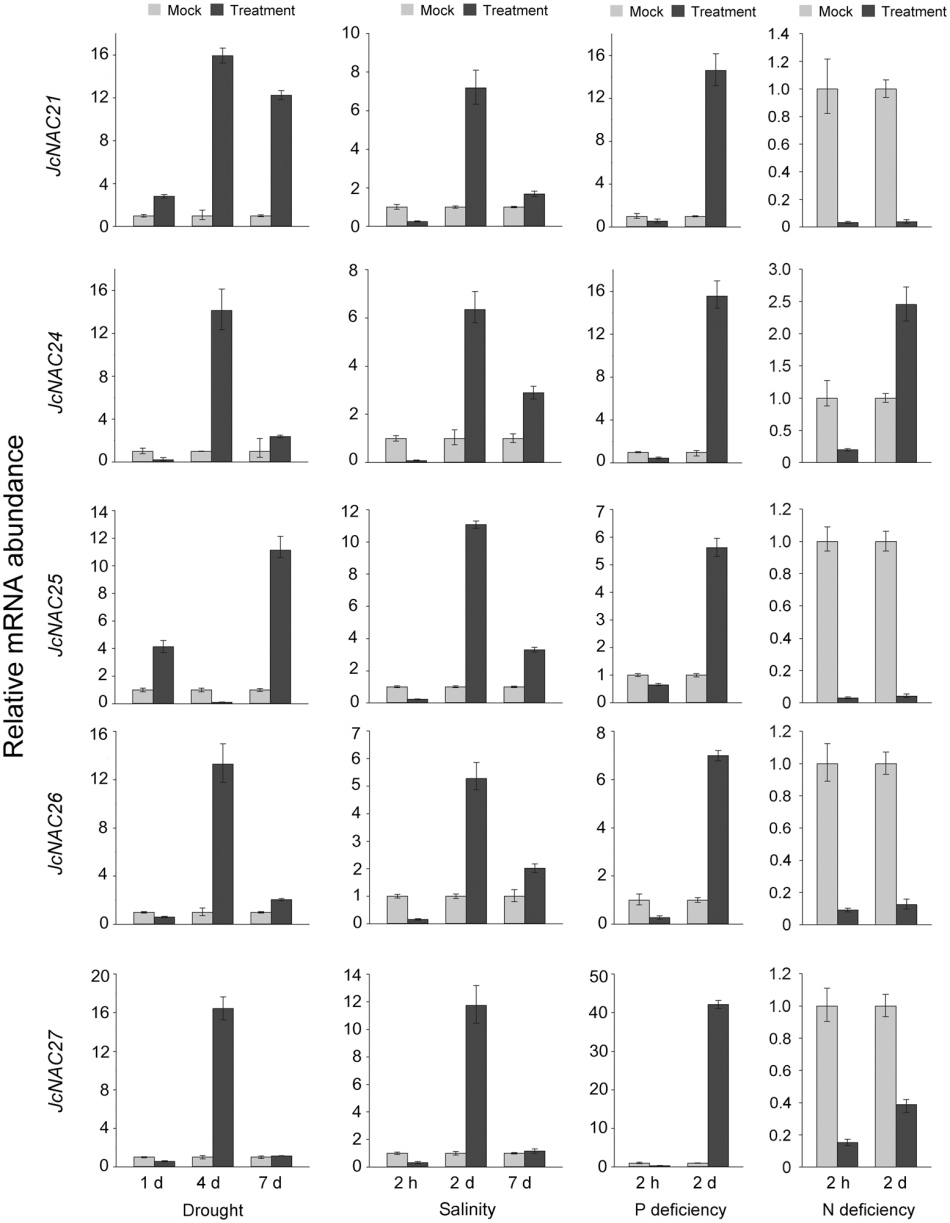
Expression analysis of selected *JcNAC* genes. Results of quantitative PCR analysis of transcripts in roots, the expression level of each gene under common condition sampled as mock. The relative expression was normalized to the reference gene *JcActin* as an internal control. The bars show standard deviations of the repeats. Each assay was run in triplicate for two independent biological repeats.

Next, we examined responses of *JcNAC* genes to four abiotic stresses (drought [42], salinity [23], phosphorus deficiency, and nitrogen deficiency) according to data retrieved from the next-generation sequencing-based digital gene expression tag database (two repeats). Differentially expressed genes (DEGs), with a greater than 2-fold difference in expression and TPM greater than or equal to 5 at one time point (and *p* value less than 0.01) were detected using Audic’s algorithm with Bonferroni Correction (http://telethon.bio.unipd.it/bioinfo/IDEG6_form/index.html) [43, 44]. Twenty-nine *JcNAC* genes were found to respond to tested abiotic stresses according to these criteria (S4 Table). Of these 29 genes, most in OG3 reportedly respond to abiotic stresses in *Arabidopsis* and rice (S3 Table) [18]. The result of our analysis indicated that six of the *JcNAC* genes in OG3 responded to the tested abiotic stresses in physic nut. To verify the digital gene expression (DGE) tag data, we selected some OG3 genes to examine the expression levels in roots after onset of drought, salinity, phosphorus and nitrogen deficiency stresses by qRT-PCR analysis (Fig 5). The results showed that *JcNAC021*, *024*, *026* and *027* genes share the same expression pattern under both drought and salinity stresses. In addition, these five genes (*JcNAC021*, *024*, *025–027*) were all upregulated under phosphorus deficiency, but downregulated (except *JcNAC024*) under nitrogen deficiency in 2 days’ roots (Fig 5). These results were generally consistent with expression changes observed in the digital gene expression tag profiling experiments.

The *cis*-acting elements control gene expression pattern in different tissues or stresses. So we analyzed the promoter regions (1 kb upstream) of all different expressed *JcNAC* genes in each treatment (drought, salinity, phosphorus deficiency, and nitrogen deficiency). The results from the PLACE program gave the known *cis*-elements which were reported in previous studies [36] (S5 Table). For example, the S000407 is the dehydration responsive element [45] presented in the regulated genes in roots exposed to drought stress (**Table A in** S5 Table), while the S000453 is the salinity-induced element [46] presented in the regulated genes in both of roots and leaves exposed to salinity stress (**Table B in** S5 Table). In addition, we predicted the new *cis*-elements of the *JcNAC* genes’ promoters using MEME program (S5 Table). It will be helpful for us to further study on the expression regulation of the *JcNAC* genes in future.

## Discussion

The NAC family genes have been classified into two major groups, Groups I and II, by comprehensive analysis of NAC sequences, and those of Group I (but not Group II) are highly conserved [7]. Group I and some Group II sequences have been further classified into 40 orthologous groups (OGs), which probably derived from a gene present in the most recent common ancestor of dicots and monocots, according to an expert NAC sequence comparison [20]. In this study, we identified a total of 100 *NAC* genes (*JcNACs*) in the physic nut genome (Table 1). After testing the predicted motifs from the JcNAC proteins, we observed that one or more motifs of the NAC domain in 40% of JcNAC proteins have diverged or been lost (Fig 1), which may have resulted in their functional divergence or nonfunctionalization. According to exon/intron structure (S2 Table) and phylogenetic analyses (S2 and S3 Figs), 60 JcNAC proteins clustered into the 39 proposed OGs for dicots, with one to five members in each OG (Table 1). These findings indicate that all the detected OGs of dicots’ *NAC* genes but OG3b have been retained in the physic nut genome.

The 15 OG5b-like (5bL) genes are close to the OG5b clade in the phylogenetic tree. Gene expansion in this group has mainly arisen from tandem duplication events. T5 on chromosome 6 and T7 on chromosome 8 contain 11 5bL tandem gene repeats (Fig 2). Nine 5bL genes cluster within the T7 locus and this duplication is also present in the castor bean genome (Fig 3 and S2 Table). Genes within this locus appear to have lost both exons and introns during their evolutionary history (Fig 1 and S2 Table). The grape genome contains multi-copy *NAC* genes in OG5b, mostly clustered on chromosome 15 (from 16, 45 Mbp to 16, 51 Mbp). This locus has at least nine *NAC* genes or pseudogenes, including *VvNAC50*-*55* (from *GSVIVT01027467001* to *GSVIVT01027477001*, http://www.phytozome.net/) [15], and two unannotated *NAC* genes between *GSVIVT01027467001* and *GSVIVT01027470001*. Two tandem *NAC* genes were annotated as a single gene of *VvNAC54* (*GSVIVT01027475001*). These results strongly suggest that 5bL genes in physic nut and castor bean evolved from OG5b genes, so we named them OG5-like (5bL). The 5bL subgroup is phylogenetically close to ANAC001 subgroup [7], which is existed in the *Populus trichocarpa* genome (within the NAC-k subgroup) [40] but not in the rice genome (**Panel G in** S3 Fig). Thirteen NAC proteins in *Arabidopsis* and six in rice contain strong *α*-helical transmembrane motifs in their C-terminus regions, some of which are involved in stress responses and phytohormone-mediated signal pathways in *Arabidopsis* [47, 48]. The genes from *Arabidopsis* and rice were distributed in OGs 4d (2 (in *Arabidopsis*), 2 (in rice)), 4g (3,1), 5b (3,2), and 5c (2, 1). The other three *Arabidopsis* genes, *ANAC001* (*AT1G01010*), *ANAC068* (*AT4G01540*), and *ANAC069* (*AT4G01550*), belong to the ANAC001 subgroup [7]. ANAC067-069 are tandem duplications. ANAC067 has lost the transmembrane motif, and may be a pseudogene [49]. Eight JcNAC proteins containing transmembrane motifs at the C-terminus were detected (S2 Table), and they were distributed in OGs 4a (1), 4d (1), 4g (1), 5b (2), 5c (1), and 5bL (2). Physic nut has two OG4d genes: *JcNAC043* and *JcNAC044*. The *JcNAC043* gene has lost two exons in its 3’-terminus, which encode the *α*-helical transmembrane motif in the *JcNAC044* gene (S2 Table). The gain and/or loss of different exons in the 3’-terminus of genes in OGs 4 and 5 has probably resulted in the gain or loss of *α*-helical transmembrane motifs in proteins encoded by these OGs during their evolution. Loss of the transmembrane motif in duplicated genes may result in functional divergence or pseudogenization in plants.

Twenty-three JcNAC proteins were assigned to four OGs. The six OG3-like (3L) genes are phylogenetically close to the OG3 clade, have higher amino acid identities in the NAC domain with OG3 proteins than other OG proteins, and the same exon/intron structure as OG3 genes. So, we propose they have the same origin as OG3 genes. The origins of *JcNAC061* and *JcNAC091* are uncertain, and they were named OGs 4, 5-like (4/5L) and OG1-like (1L), mainly based on their exon/intron structures and the phylogenetic analysis. The orthologs of 3L genes were detected in subgroups of NAC-j and NAC-m, while orthologs of 1L genes were in the NAC-n subgroup of *Populus NAC* genes [40]. The ortholog of 4/5L genes was detected in the genome of *T*. *cacao* (its locus names in Phytozome (http://www.phytozome.net/) is Thecc1EG033799), but not in *Populus*, *Arabidopsis* and rice. On the other hand, a number of *NAC* genes containing one of two introns within their NAC domain coding sequences in *Arabidopsis* and rice were unclassified into the determined OGs (**Panel G in** S3 Fig). The amino acid sequences of their NAC domains are highly divergent from other subgroup NAC proteins, and their orthologs were not found in the physic nut genome. These results indicate that different plant linkages may have either lost or acquired some of the NAC subfamilies during their evolution. Among these genes, only *ANAC095* (*AT5G41090*.*1*) was reported to play a role in male gametophyte development in *Arabidopsis* as yet [50].

Intronless (IL) *NAC* genes were found in all examined plants, and they fall into the previously reported Group II [7]. The IL genes from different species were clustered into distinct subgroups among different plant groups (S2 Fig and **Panel G in** S3 Fig). Seven IL *NAC* genes were detected in physic nut, and 13 in castor bean. Most of them are tandem duplications. In physic nut, the IL genes are clustered in T2 and T10 on chromosomes 4 and 10 (Fig 2), while scaffold 29657 of castor bean contains a cluster of six IL *NAC* genes. *Arabidopsis* also has a cluster of five IL *NAC* genes (*AT1G60280*, *AT1G60300*, *AT1G60340*, *AT1G60350*, and *AT1G60380*). The origin of these IL *NAC* genes is unclear because their NAC domains appear to be very divergent from the other NACs and they do not have conserved amino acid sequences among different plant groups. Furthermore, little is known about their functions in plants as yet.

The only plant species of the seven examined (physic nut, *Arabidopsis*, castor bean, grape, rice, poplar, and soybean) in which single intron-containing (SI) genes were detected was physic nut. In this group, NAC subdomains A-D are encoded by the first exon, and subdomain E by the second exon (S2 Table). Using these proteins as queries in BlastP searches against public protein databases, we found orthologs in the *T*. *cacao* genome (Thecc1EG022059, Thecc1EG030287, Thecc1EG036193, Thecc1EG036197, and Thecc1EG036236). These results indicate that many plant species have lost SI genes during their evolution. Their origin is also unclear because their NAC domains appear to be very divergent from those of other NACs.

The segmental duplication putatively associated with the salicoid genomic duplication event appears to have significantly contributed to the amplification of many multi-gene families [31]. Accordingly, our phylogenetic analysis and chromosomal mapping indicate that segmental duplication blocks (A) contain some of the duplicated genes (of the same OGs) in the physic nut genome (Fig 2) based on the corresponding duplicate genomic blocks established in this species [26], e.g. OGs 3c and 8b in A1 blocks, OG7a in A2 blocks, OG3c and 5bL in A3 blocks, 5bL in A4 blocks, and SI in A5 blocks. Tandem duplications may also have affected expansion of the *NAC* gene family [14–16, 40]. The gene loci analysis revealed the presence of 11 tandem duplicate clusters (T), including 30% of the *NAC* genes in the physic nut genome. Genes in eight of these clusters were classified into the same OGs. However, three of these tandem duplications could be ancient, resulting in divisions of the duplicated genes into different clades in the phylogenetic tree. As mentioned above, several tandem duplicates in the T7 locus appear to have lost or gained exons, and may have undergone pseudogenization like their orthologs in *Arabidopsis* [49]. The T6 tandem duplicates probably arose from an ancient duplication event preceding the divergence of monocots and dicots [20]. T1 tandem duplications likely arose from a dicot-specific event that divided OGs 3e and 3d, as OG3e has not been detected in monocots (Table 1; Fig 2 and S2 Fig). Only T4, T8, T9, and T11 tandem duplications likely occurred after separation of lineages of the analyzed species (Fig 2). Additionally, several duplicates from the ancient duplication events (A1, A2, and A3) and tandem duplication events (T7 and T11) show divergent expression patterns (Fig 4 and S4 Table), suggesting the occurrence of subfunctionalization during the evolutionary process.

The function of *NAC* genes in most OGs has been addressed in *Arabidopsis* and/or other plants (S3 Table) [20]. We found that many NAC orthologs of physic nut and *Arabidopsis* have similar tissue-specific, and abiotic stress response, expression patterns (S3 Table). For example, the OG3d genes *ATNAC2/ANAC056* and *ANAC018* in *Arabidopsis* participate in regulation of embryogenesis [51]; while in physic nut *JcNAC028* and *029* are only highly expressed in seeds. Most *ANAC* genes in OG3 respond to abiotic stresses in *Arabidopsis* [39]. For instance, the OG3d gene *ATNAC2* in *Arabidopsis* reportedly responds to salinity stress, but not drought stress [49], and the same responses were observed for *JcNAC028* and *029* in physic nut roots (S3 Table). The expression levels of OG3e genes (*ANAC01*, *ATNAC3/ANAC055* and *JcNAC021*) as well as 3c genes (*ANAC029*, *ANAC047*, *JcNAC026* and *JcNAC027*) are upregulated under drought and salinity stresses in both *Arabidopsis* [52] and physic nut (Fig 5 and S4 Table). Expression levels of OG3a genes (*ATAF1*/*ANAC002*) are upregulated under both drought and salinity stresses and play negative roles in abiotic stress resistance *Arabidopsis* [53, 54]. The expression of *JcNAC021*, *024*, *026* and *027* are also reportedly upregulated under salinity stress in roots and leaves of physic nut seedlings [23]. These results imply that most *JcNAC* genes in physic nut have similar functions to their orthologs in *Arabidopsis*, but several may have functionally diverged from their orthologs.

In addition, the expression of *JcNAC021*, *024*, *025*, *026* and *027* respond to phosphorus and nitrogen deficiency (Fig 5). There are few reports on responses to this stress in other species. So these results may help efforts to identify further roles of NAC family members. No credible expression of the SI genes was observed in physic nut based on the available transcript sequences and next-generation sequencing-based digital gene expression tags. However, they may be expressed in other tissues (e.g. ESTs representing four orthologous SI *NAC* genes appear to be expressed in *J*. *integerrima* flowers), or some of them may be pseudogenes.

In conclusion, we identified a total of 100 *JcNAC* genes in the physic nut genome. Sixty of the proteins they putatively encode were grouped into 39 previously predicted OGs of *NAC* gene family. About 30% of the *JcNAC* genes were replicates, clustered at several locations in the physic nut genome, apparently originating from both ancient and recent duplication events. Tissue-specific expression profiles of JcNAC transcripts under normal growth conditions were examined, and 29 *JcNAC* genes were also found to respond to tested abiotic stresses. The new *JcNAC* gene sequences and expression information reported here should facilitate further investigations of the functions of *NAC* genes in plant development and stress responses, particularly in physic nut.

## Supporting Information

S1 Fig Motif logos and subdomain compositions of JcNAC proteins. Motif logos were obtained from MEME (http://meme.nbcr.net/meme/) [35]. The height of a letter indicates its relative frequency at the given position. Motifs 1 plus 8, 4, 6 plus 2, 5 plus 3, and 7 were identified as NAC subdomains A to E, respectively.
(TIF)

S2 Fig Maximum likelihood phylogenetic tree of NAC proteins. Phylogenetic analysis was conducted with protein sequences from *A*. *thaliana*, *O*. *sativa*, *R*. *communis* and *J*. *curcas*. Branch support values correspond to approximate likelihood ratio test (a-LRT) results. Bootstrap scores higher than 50% are indicated on the nodes. The 40 distinct orthologous groups (OGs) are indicated by blue characters with the names assigned by Cenci et al. [20]. We named the OGs marked by green characters. Detailed grouping information is given in S3 Table.
(TIF)

S3 Fig Maximum likelihood phylogenetic trees of each main cluster of NAC proteins. (PDF)

S1 Table Primers used for qRT-PCR. (XLS)

S2 Table Overview of the *JcNAC* genes. (XLS)

S3 Table List of the *NAC* gene family based on phylogenetic analysis in this study. (XLS)

S4 Table The expression of *JcNAC* genes based on digital gene expression analysis. (XLS)

S5 Table Putative *cis*-acting elements enriched in promoters of *JcNAC* genes exposed to four different stresses. (XLS)
