# Supplementary figures and images for: Genome-Wide Analysis of the NAC Gene Family in Physic Nut (Jatropha curcas L.)
Source: PLoS One. 2015 Jun 30;10(6):e0131890. doi: 10.1371/journal.pone.0131890 (PMC4488383; doi:10.1371/journal.pone.0131890)

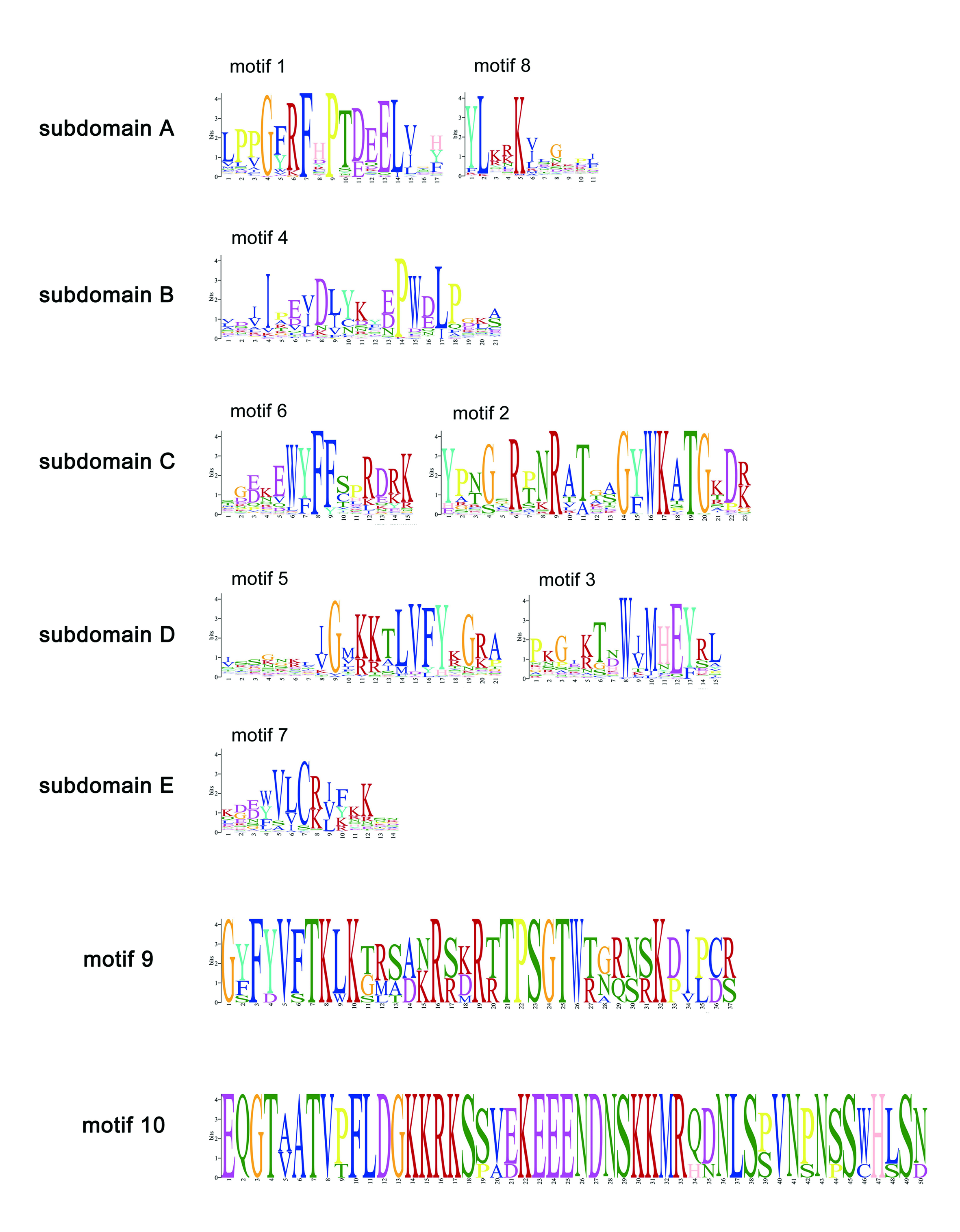

Supplement: S1 Fig — Motif logos were obtained from MEME (http://meme.nbcr.net/meme/) [35]. The height of a letter indicates its relative frequency at the given position. Motifs 1 plus 8, 4, 6 plus 2, 5 plus 3, and 7 were identified as NAC subdomains A to E, respectively. (TIF) [file pone.0131890.s001.tif]

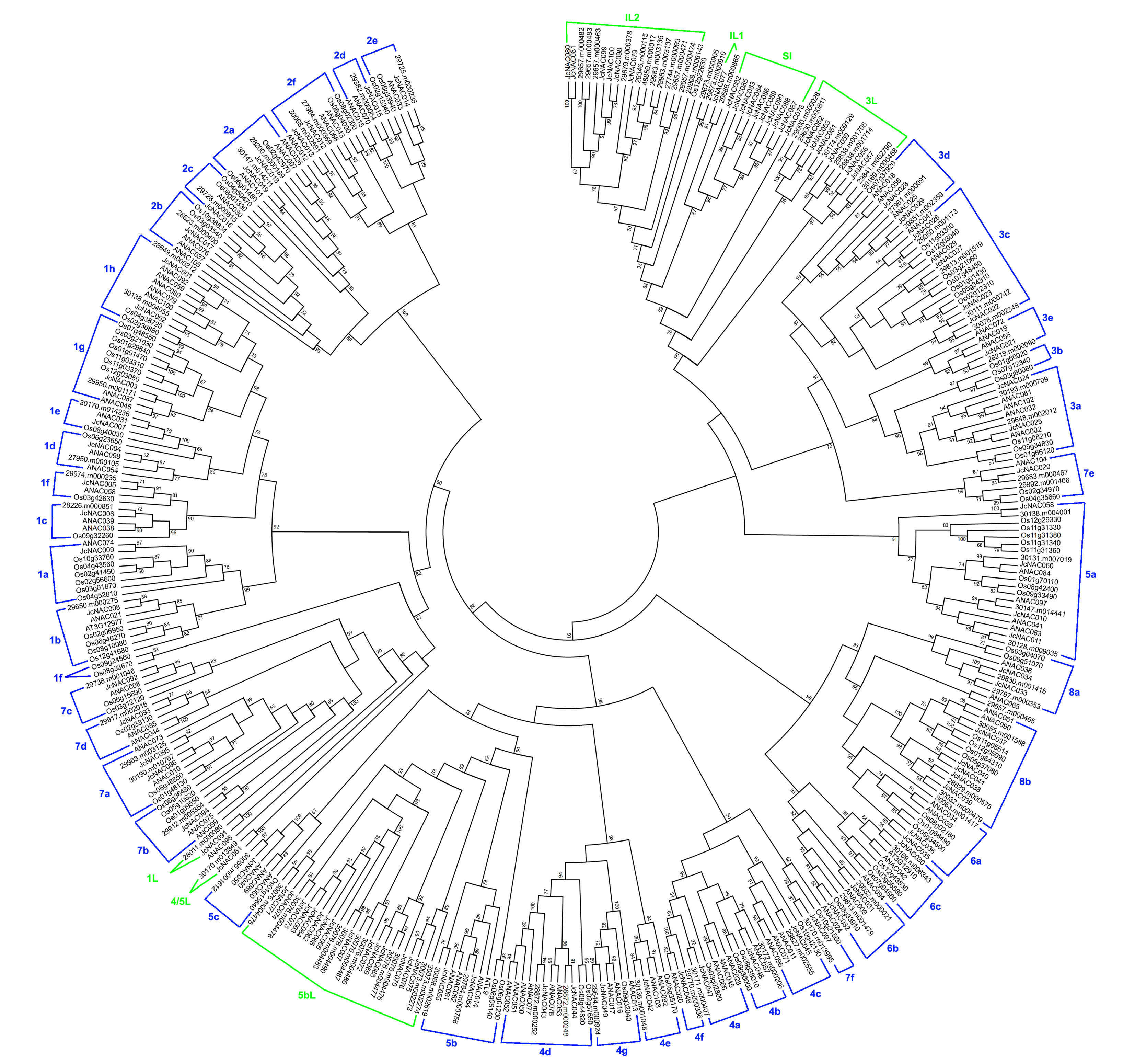

Supplement: S2 Fig — Phylogenetic analysis was conducted with protein sequences from A. thaliana, O. sativa, R. communis and J. curcas. Branch support values correspond to approximate likelihood ratio test (a-LRT) results. Bootstrap scores higher than 50% are indicated on the nodes. The 40 distinct orthologous groups (OGs) are indicated by blue characters with the names assigned by Cenci et al. [20]. We named the OGs marked by green characters. Detailed grouping information is given in S3 Table. (TIF) [file pone.0131890.s002.tif]
