# Supplementary material for: Genome-Wide Analysis of the NAC Gene Family in Physic Nut (Jatropha curcas L.)
Source: PLoS One. 2015 Jun 30;10(6):e0131890. doi: 10.1371/journal.pone.0131890 (PMC4488383; doi:10.1371/journal.pone.0131890)

S3 Fig. Maximum likelihood phylogenetic trees of each main cluster of NAC proteins.

Panel A. CL00003: OGs 1a-h

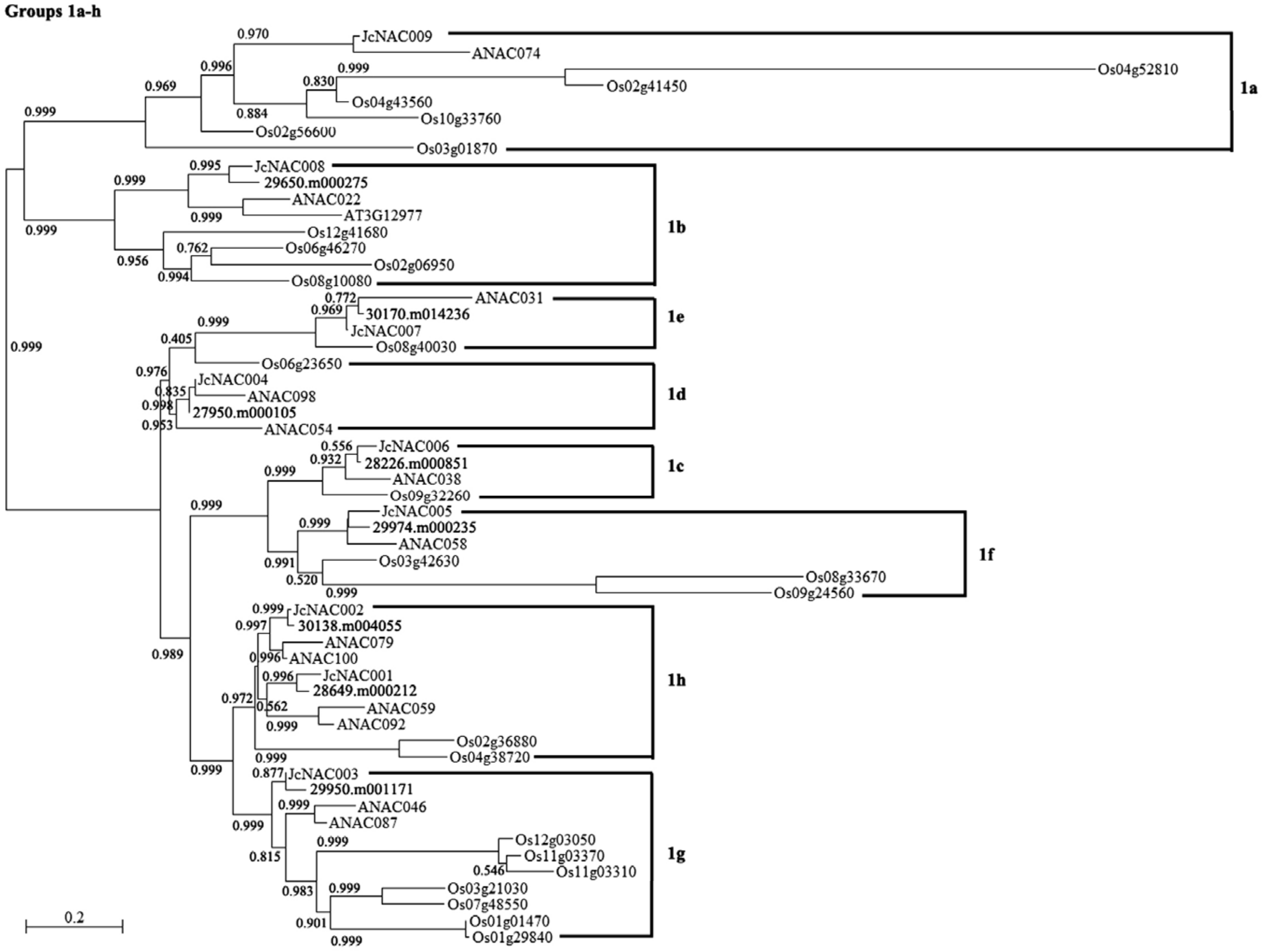

Panel B. CL000006: OGs 2a-f

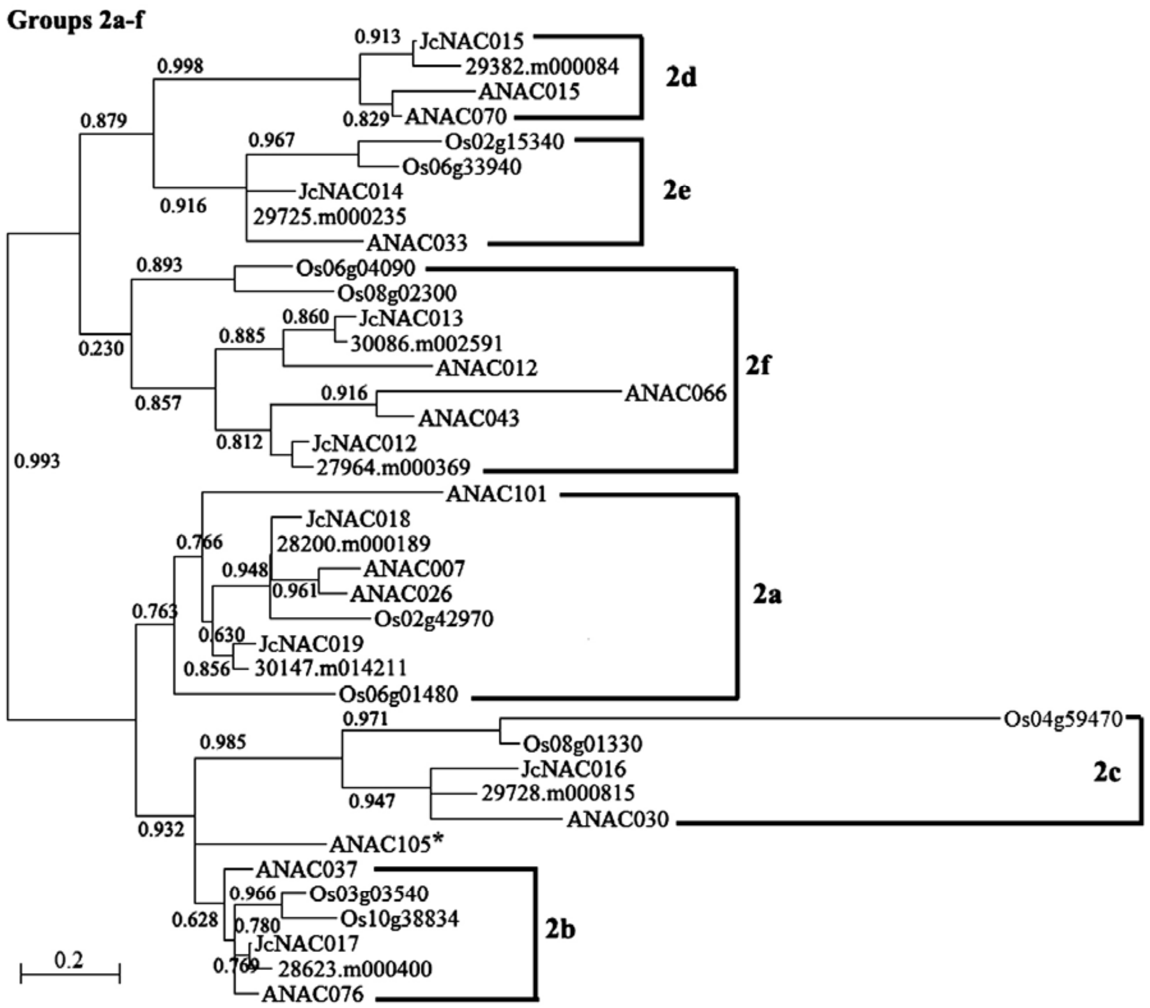

Panel C. CL000002: OGs 3a-e, 5a, 7e

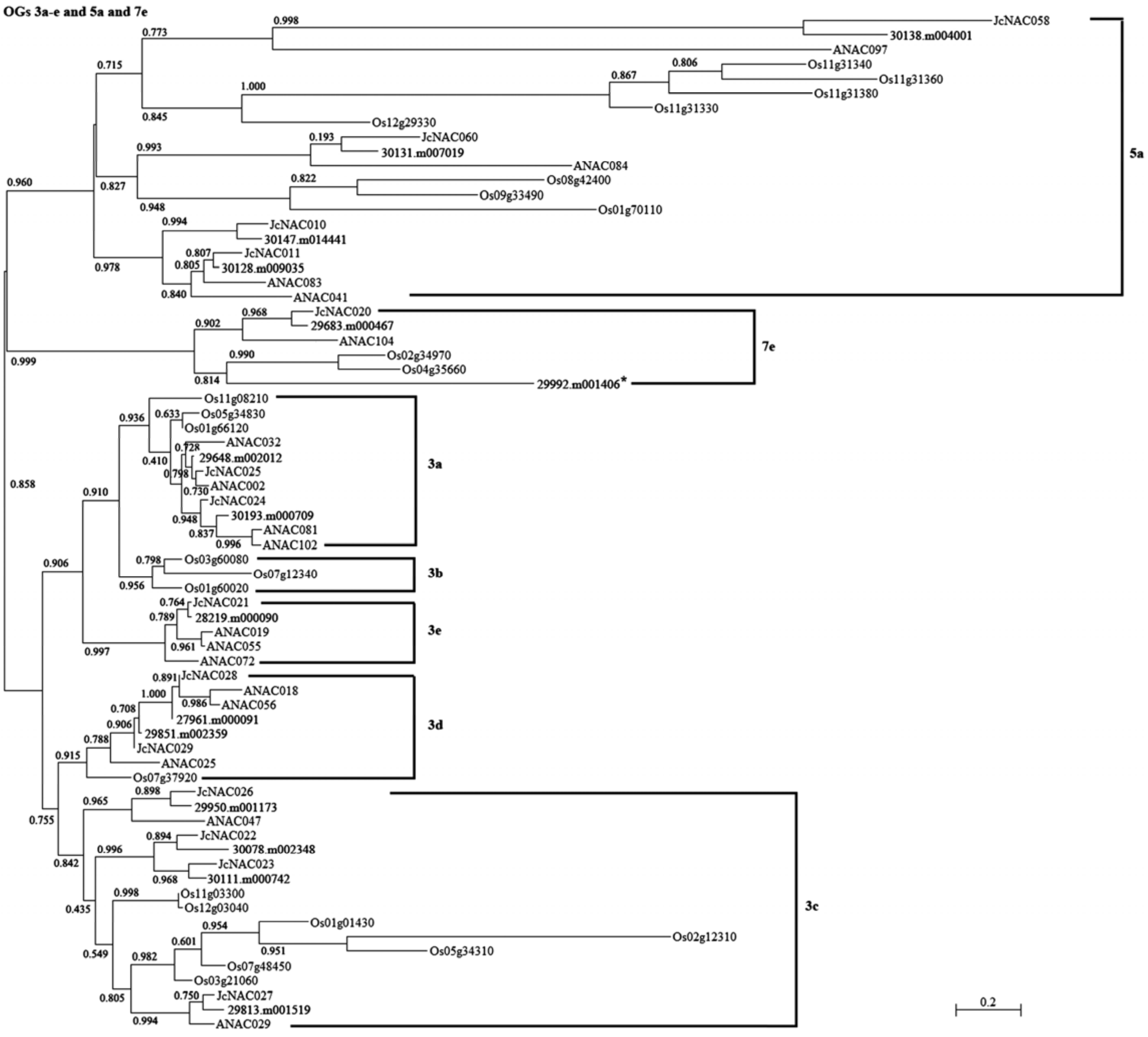

Panel D. CL000004: OGs 4a-g, 5b-c

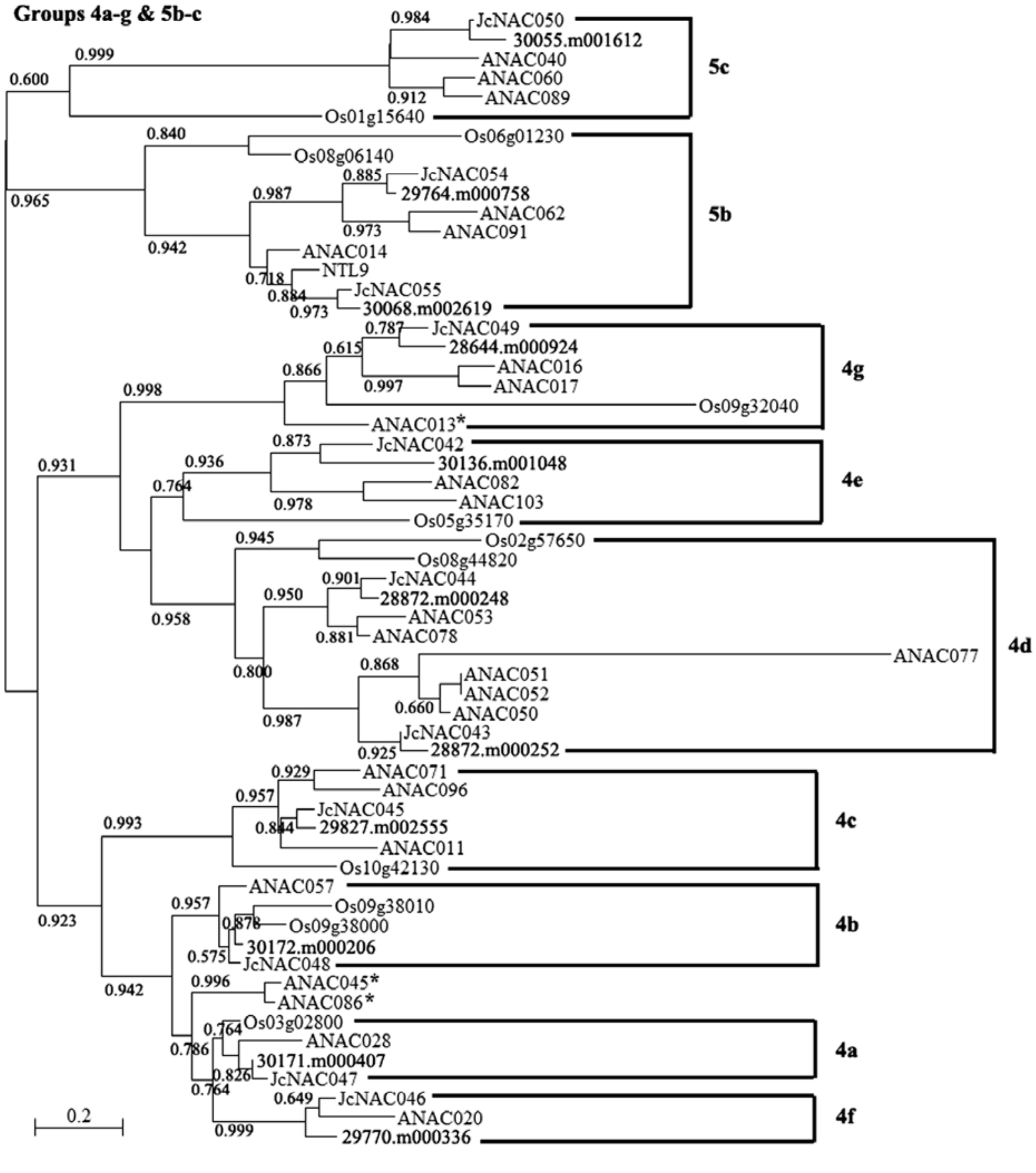

Panel E. CL000005: OGs 6a-c, 8a-b

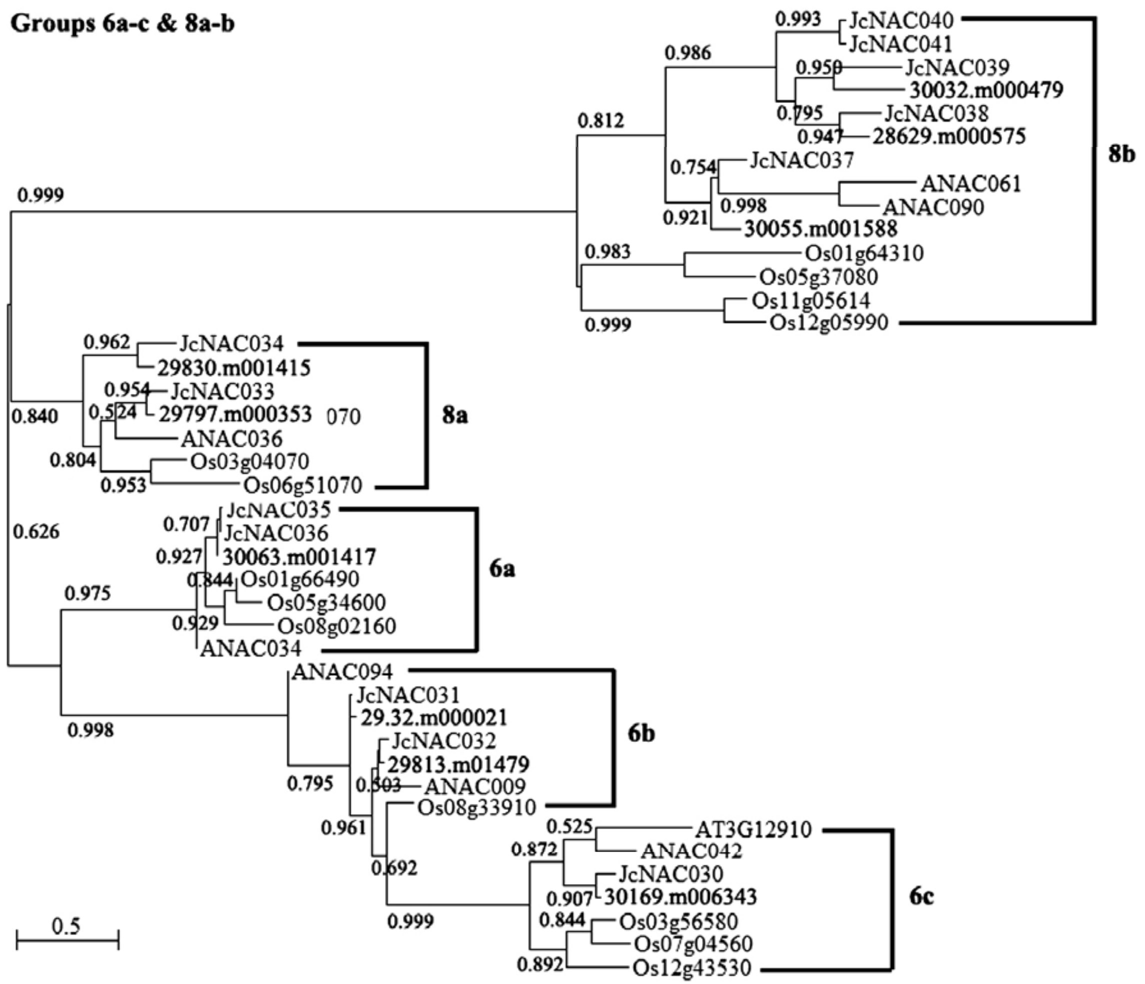

Panel F. CL000007: OGs 7a-d

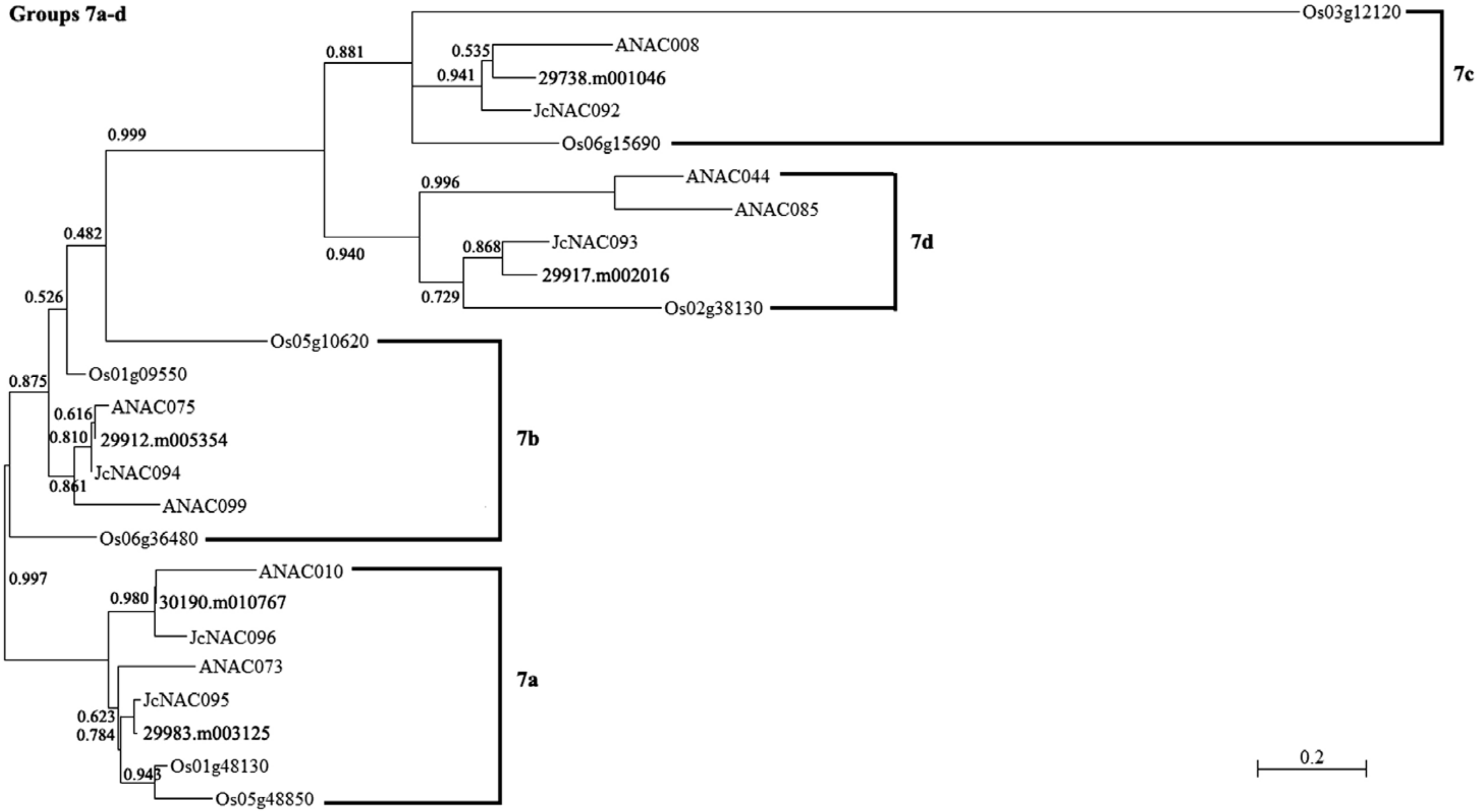

Panel G. Phylogenetic tree of the unclassified NAC proteins from physic nut, Arabidopsis and rice

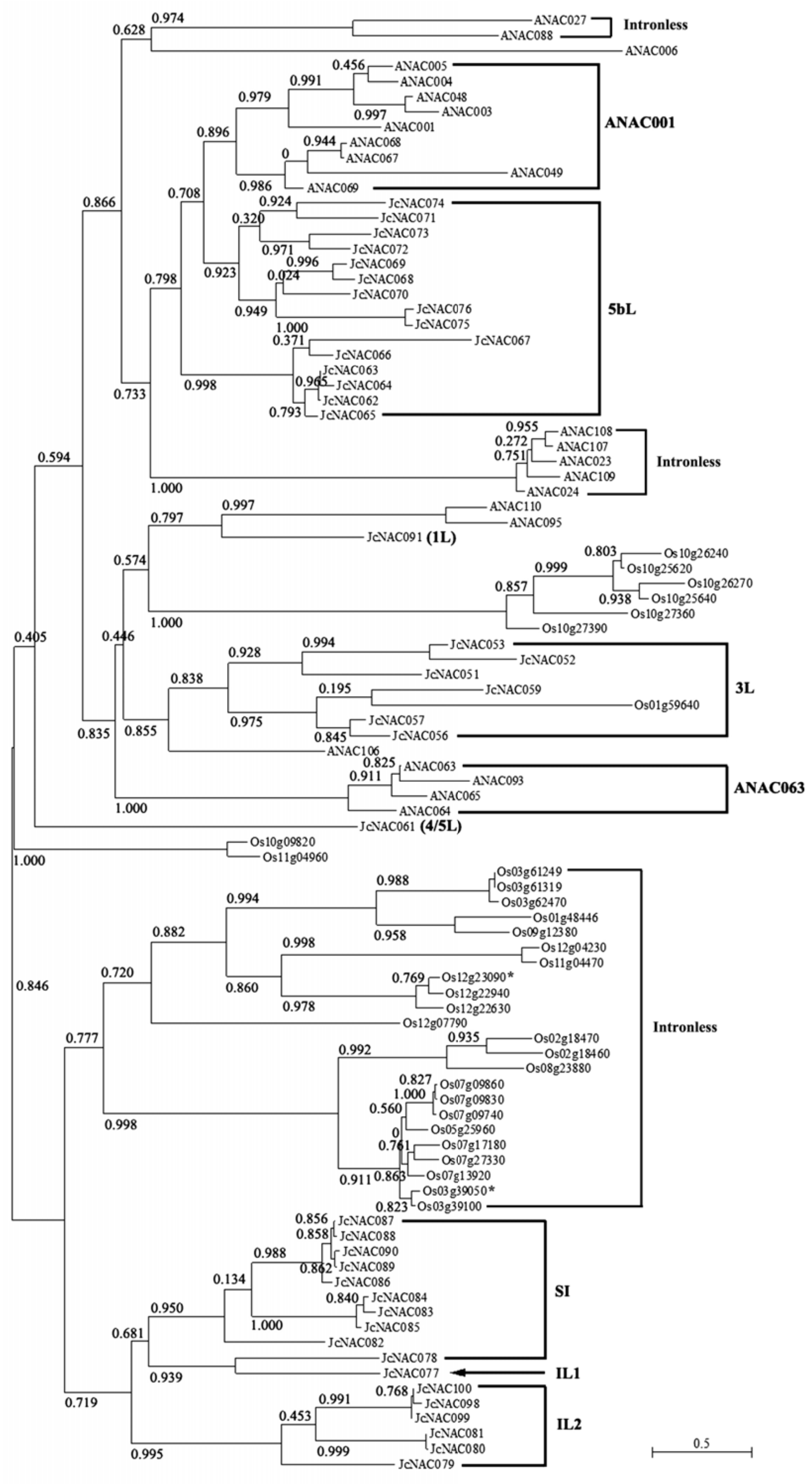

Supplement: S3 Fig — (PDF) [file pone.0131890.s003.pdf]
